# Retracted Publications in Otolaryngology–Head and Neck Surgery: What Mistakes Are Being Made?

**DOI:** 10.1002/oto2.157

**Published:** 2024-06-13

**Authors:** Hannaan S. Choudhry, Sugosh M. Anur, Hassan S. Choudhry, Emily M. Kokush, Aman M. Patel, Christina H. Fang

**Affiliations:** ^1^ Department of Otolaryngology–Head and Neck Surgery Rutgers New Jersey Medical School Newark New Jersey USA; ^2^ Department of Otolaryngology–Head and Neck Surgery Philadelphia College of Osteopathic Medicine Philadelphia Pennsylvania USA; ^3^ Department of Otolaryngology–Head and Neck Surgery Albert Einstein School of Medicine/Montefiore Medical Center Bronx New York USA

**Keywords:** bibliometric analysis, Otolaryngology, publications, retractions, withdrawn

## Abstract

**Objectives:**

Retraction of publications is critical to maintaining scientific integrity, yet there is a lack of research on its occurrence in Otolaryngology. This study investigates characteristics, trends, and reasons for retraction of publications in otolaryngology journals.

**Study Design:**

Bibliometric analysis.

**Setting:**

PubMed, Scopus, Web of Science.

**Methods:**

A PubMed search for publications retracted during 1990 to 2022 from the top 60 journals with the subject “Otorhinolaryngology” using Scopus' CiteScore was performed. Publications were excluded if they were not in English, had missing information or did not have available abstracts or full‐text. Publication and retraction dates, journal, country of origin, citation counts, journal impact factor (JIF), topic, and reason for retraction were recorded. Pearson correlation coefficients were calculated to identify potential associations in the data.

**Results:**

Fifty‐three publications were included. The 2020s had the highest number of retractions per year (4.33), with publications being retracted on average, 35 months after initial publication. The most common retracted topic and country of origin were head and neck (26.4%) and China (17.0%), respectively. Most publications were retracted because of plagiarism or duplicate publication (52.8%). Mean citation count was 6.92 ± 8.32 and mean JIF was 2.80 ± 1.35. Citation count was positively associated with months until retraction (*r* = .432, *P* = .001). There was no significant correlation between months to retraction and JIF (*r* = .022, *P* = .878).

**Conclusion:**

The most cited reasons for retraction were plagiarism and duplicate publication. An understanding of the reasons for retraction can better position journals to enforce more meticulous review standards and reduce such publications from being published.

**Level of Evidence:**

Level 4.

In scientific writing, cases of both intentional and unintentional misconduct or error are known as retractions. While some believe that retractions occur in less than 1 out of every 5000 publications, empirical evidence has supported that retraction rates have increased since the start of the 21st century.^1^ Due to journals having inconsistent retraction methods in the past, the Committee of Publication Ethics (COPE) created a set of guidelines on handling retractions in 2009.^2^ These guidelines state that journal editors should consider retracting publications if there is clear evidence of unreliable findings (due to misconduct or honest error), plagiarism, or unethical research. In cases of an honest error in a small portion of an otherwise reliable publication, editors can consider issuing a correction if the small portion is misleading.^3^ Retraction notices should be linked to the original publication, clearly indicate the reason for retraction, identify who is retracting the publication, and be freely available to all readers.^3^ Since the potential implications of flaws in reputable journals can be serious, publications are retracted of their published status in order to have these flaws corrected.^1^ In more extreme cases of scientific fraud, retracted publications are deservedly stripped of the opportunity to be published in the future.

Previous studies have shown that most retracted scientific publications can be attributed to cases of fraud, duplicate publication, and plagiarism.^4^ Even with peer‐review methods in place that evaluate the quality of submitted literature, there are still cases of retraction. While this may point to a need to improve the quality and consistency of peer‐review processes among journals, the authors of retracted publications themselves should be held accountable for numerous potential unethical motives, including conflict of interest and financial implications.^5^ One instance is a publication by Schubert and Derr in 1979 on the removal of toxic chemicals in the blood, which amounted to nearly $3 million of fraudulently earned research grants.^5^ Not only do cases of retractions result in financial losses, but they also limit opportunities for others to conduct authentic research that can eventually get published in top scientific journals.

Most importantly, higher occurrences of scientific misconduct in medicine can potentially result in worse patient care due to misleading methodology and results.^5^ Therefore, more research must be done on the retraction of publications in medicine. While retraction studies have already been performed in General Surgery, Ophthalmology, Orthopedics and Neurosurgery, there has not yet been sufficient research conducted in Otolaryngology–Head and Neck Surgery (OHNS).^6^, ^7^, ^8^, ^9^ The purpose of this study is to gather former publications in OHNS that were ultimately retracted and determine the causes and characteristics of these retracted publications.

## Methods

The top 60 journals with the subject of “Otorhinolaryngology” were determined by highest 2021 CiteScores on Scopus Sources (https://www.scopus.com/sources.uri) (Supplemental Table S1, available online). On January 2, 2023, a search was conducted in PubMed to identify publications in each journal that were retracted during the time period January 1, 1990, to December 31, 2022. The search query used the syntax: (“Journal Name”[Journal]) AND ((“retracted publication”[Publication Type]) OR (“retraction of publication”[Publication Type])). This search strategy was applied to all journals of interest until all retracted publications were compiled. Publications were excluded if the abstract and full‐text were not in the English language or did not have abstracts or full‐text available. Data were extracted from publications based on methodology from similar papers.^6^, ^7^, ^8^, ^9^ To identify the total number of publications in these 60 journals during the study time period, a PubMed search query using the syntax: (“Journal Name”[Journal]) was performed from 1990 to 2022. H.S.C. and S.M.A. independently performed data extraction. Any discrepancies in the data extraction were resolved by C.H.F. if a consensus between the 2 data extractors could not be reached. The data obtained include original publication's initial Epub date, primary author, country of origin, journal impact factor (JIF), subspecialty topic of paper (Basic Science, Facial Plastics and Reconstruction, Head and Neck, Laryngology, Oral and Maxillofacial Surgery, Otology/Neurotology, Pediatrics, Rhinology, Sleep, Other), study type (Anatomical, Case Report, Clinical Trial [randomized and nonrandomized], Experimental, Laboratory Research, Observational, Oral Presentation, Prospective, Retrospective, Review Article, Systematic Review/Meta‐Analysis), number of citations, reason for retraction (Author Misattribution, Data Error, Duplicate, Formatting Error, Fraudulent Data, Lack of Ethics Approval/Consent, Misconduct, Plagiarism, Multiple Reasons, Other, Unknown), and date of retraction. Time to retraction was calculated by the time in years between the date of original publication and retraction. JIFs and citation count were determined using *Journal Citation Reports* (*JCR*) from Web of Science.

All data were recorded in Microsoft Excel and exported for analysis to SPSS Statistics Version 25.0 (IBM Corporation). Descriptive statistics on the retracted publications were reported. Additionally, the relationships between time to retraction, citation count, and JIF were evaluated separately using Pearson correlation coefficients and *P* values derived from the Pearson correlation test. A *P* value less than .05 was considered statistically significant. Institutional review board (IRB) approval was not required for this review because it qualifies as nonhuman subject research according to the protocols set forth by the IRB of Rutgers New Jersey Medical School (Newark, NJ).

## Results

**Figure 1:**
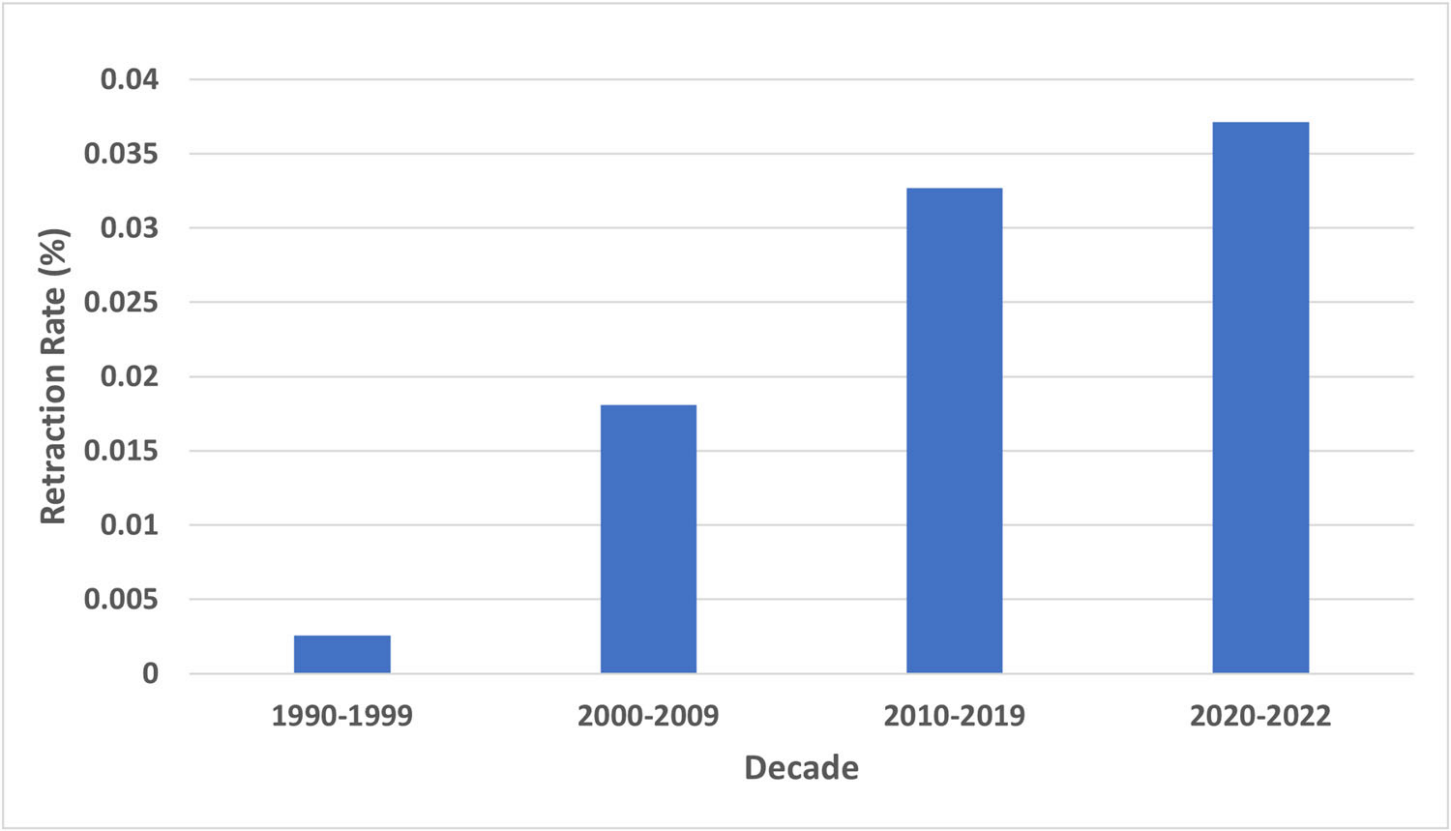
Retraction rates in each decade during 1990 to 2022 for Otolaryngology–Head and Neck Surgery publications in the top 60 Otorhinolaryngology journals according to 2021 Scopus rankings. Data were calculated by dividing the number of retracted publications from these journals by the total number of publications in these journals as found in PubMed.

**Figure 2:**
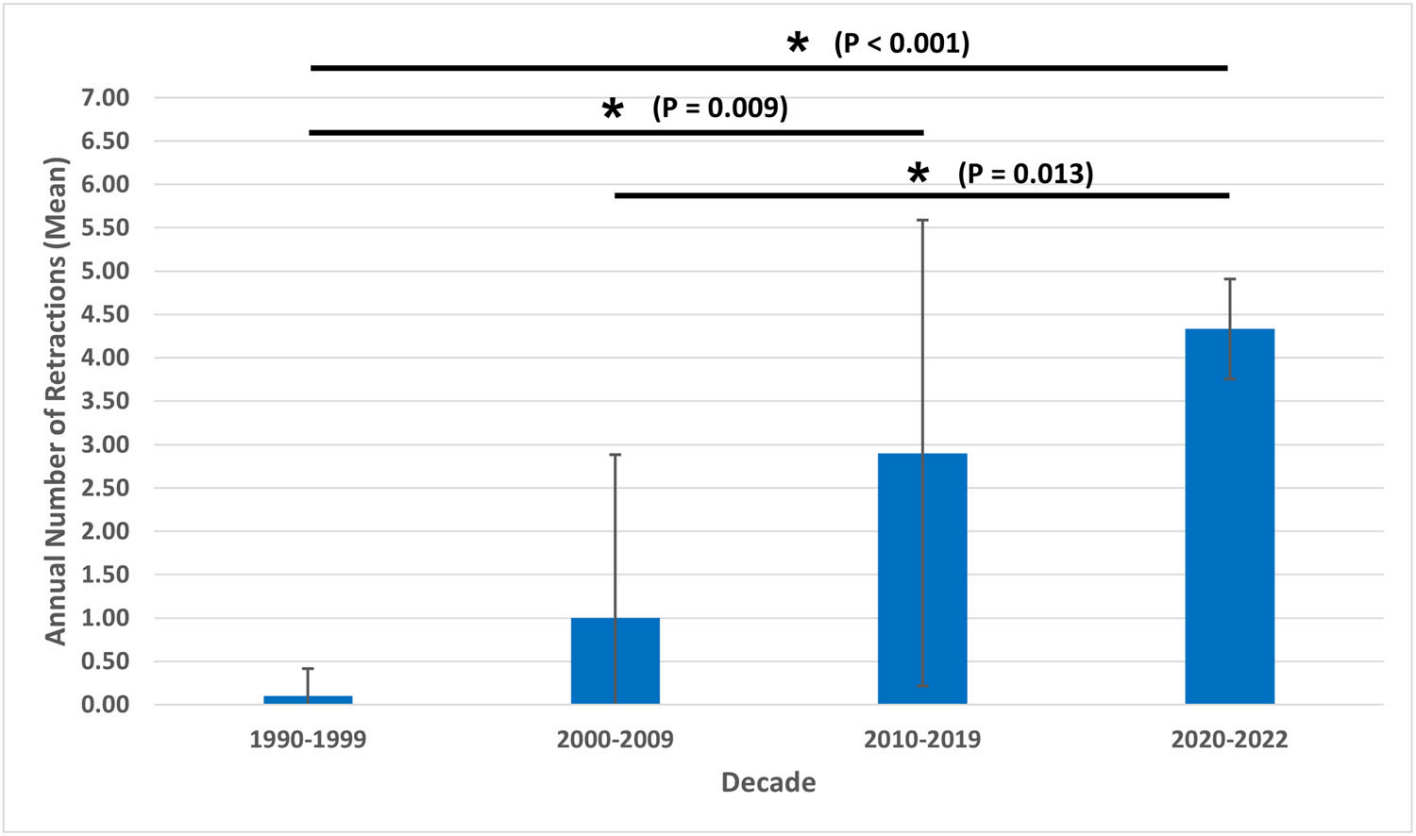
Average number of annual retractions classified by decade during 1990 to 2022 for Otolaryngology–Head and Neck Surgery publications in the top 60 Otorhinolaryngology journals according to 2021 Scopus rankings. *A statistically significant difference in the average number of annual retractions between the 2 decades (*P* < .05).

A total of 98 publications that were retracted during 1990 to 2022 were identified using our initial search query. After screening for duplicates, title/abstract, and full‐text publications using the inclusion and exclusion criteria, 53 publications were included for analysis (Supplemental Table S2, available online). The retraction rate for publications in the included journals increased each decade, rising to 0.037% during the 2020 to 2022 timeframe (Figure 1). The average annual number of retractions increased with time period, with the majority of annual retractions occurring between 2020 and 2022 (4.33) and the least occurring between 1990 and 1999 (0.10) (Figure 2). The average number of retractions during 2020 to 2022 was statistically significantly greater than the number during 1990 to 1999 (*P* < .001) and 2000 to 2009 (*P* = .013), and the average number of retractions during 2010 to 2019 was statistically significantly greater than the number during 1990 to 1999 (*P* = .009).

### Journals and Authors

The 53 studies originated from journals with impact factors (IFs) ranging from 1.22 to 8.96, with an average IF of 2.80. Authors publishing retracted literature originated from 1 of 21 total countries. The majority of authors originated from China (17.0%), followed by the United States (15.1%), Japan (11.3%), India (7.5%), and the United Kingdom (7.5%) (Table 1). Two authors were listed as primary authors for multiple retracted publications (2 and 6, respectively). An average of 6.92 other publications cited each retracted publication (range: 0‐38). Comparison between this number of publications citing each retracted publication and the associated JIF for each retracted publication showed a significant correlation (*r* = .425, *P* = .002).

**Table 1:** Country of Origin of Retracted Otolaryngology–Head and Neck Surgery Publications in Top 60 Journals From 1990 to 2022

Country of origin	N (%)
China	9 (17.0)
United States	8 (15.1)
Japan	6 (11.3)
India	4 (7.5)
United Kingdom	4 (7.5)
Australia	2 (3.8)
Hungary	2 (3.8)
Iran	2 (3.8)
Italy	2 (3.8)
South Korea	2 (3.8)
Sweden	2 (3.8)
Brazil	1 (1.9)
Canada	1 (1.9)
Egypt	1 (1.9)
Germany	1 (1.9)
Libya	1 (1.9)
Mexico	1 (1.9)
Saudi Arabia	1 (1.9)
Taiwan	1 (1.9)
Thailand	1 (1.9)
Turkey	1 (1.9)
Total	53 (100.0)

### Study Topic and Type

The publications in our study were categorized into 11 different categories based on subspecialty topic. The most common topic for retracted publications was Head and Neck (26.4%), followed by Otology/Neurotology (15.1%) and Oral and Maxillofacial Surgery (13.2%) (Table 2). The topic with the least number of retracted publications in our study was Sleep (1.90%). The publications were of various study types with the majority being clinical trials (32.1%), followed by review articles (15.1%), retrospective studies (13.2%), and case reports (13.2%) (Table 3).

**Table 2:** Primary Topic for Retracted Otolaryngology–Head and Neck Surgery Publications in Top 60 Journals From 1990 to 2022

Topic	N (%)
Head and Neck	14 (26.4)
Otology/Neurotology	8 (15.1)
Oral and Maxillofacial Surgery	7 (13.2)
Laryngology	5 (9.4)
Other	5 (9.4)
Facial Plastics and Reconstruction	4 (7.5)
Rhinology	4 (7.5)
Basic Science	3 (5.7)
Pediatrics	2 (3.8)
Sleep	1 (1.9)
Total	53 (100.0)

**Table 3:** Type of Publication for Retracted Otolaryngology–Head and Neck Surgery Publications in Top 60 Journals From 1990 to 2022

Publication type	N (%)
Clinical trials	17 (32.1)
Review article	8 (15.1)
Case reports	7 (13.2)
Retrospective study	7 (13.2)
Systematic reviews/meta‐analyses	3 (5.7)
Observational studies	3 (5.7)
Laboratory research	3 (5.7)
Prospective study (unspecified)	2 (3.8)
Anatomical study	1 (1.9)
Experimental	1 (1.9)
Oral pesentation	1 (1.9)
Total	53 (100.0)

### Retractions

**Figure 3:**
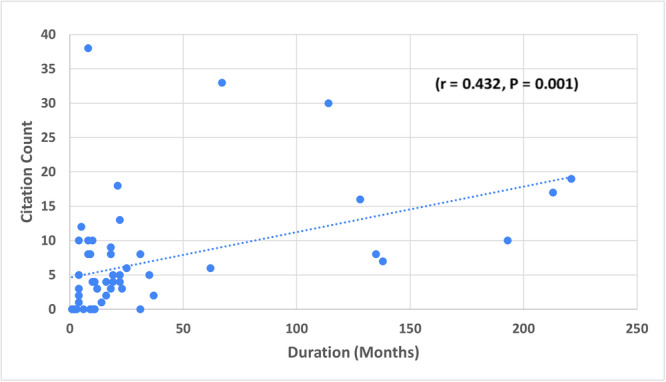
The number of months to retraction for each retracted publication during 1990 to 2022 in the top 60 Otorhinolaryngology journals according to 2021 Scopus rankings versus the number of times each retracted publication was cited by other publications.

OHNS studies in our research were categorized into 11 different reasons for retraction. All the studies in our analysis had retraction notifications on PubMed or on the individual journal's website. The most frequent reason for retraction was plagiarism (28.3%), followed by duplicate publication (24.5%), data error, consisting of mistakes in methodology or analysis (9.40%), fraudulent data (7.5%), and lack of ethical approval/consent (7.5%) (Table 4). Authors were notified of the decision to retract the publication from its respective journal in a variable timeframe ranging between 1 and 221 months, with an average notice time of 35.04 months. The time to notification of a retracted publication status was significantly positively correlated with the number of times a publication was cited (*r* = .432, *P* = .001), however, no correlation existed with the journal's IF (*r* = .022, *P* = .878) (Figure 3).

**Table 4:** Cited Reasons for Publication Retraction for Retracted Otolaryngology–Head and Neck Surgery Publications in Top 60 Journals From 1990 to 2022

Reason	N (%)
Plagiarism	15 (28.3)
Duplicate	13 (24.5)
Data error	5 (9.4)
Fraudulent data	4 (7.5)
Lack of ethics approval/consent	4 (7.5)
Formatting error	3 (5.7)
Unknown	3 (5.7)
Author misattribution	2 (3.8)
Misconduct	2 (3.8)
Multiple reasons provided	1 (1.9)
Other	1 (1.9)
Total	53 (100.0)

## Discussion

The sheer number of papers published today across medical disciplines is unmatched compared to any timeframe of the past. However, previous literature across multiple surgical fields has shown that the number of retracted publications has increased over time as well.^6^, ^7^, ^8^, ^9^, ^10^ Furthermore, a recent study found that OHNS was fourth in the absolute number of retracted publications among surgical subspecialty research, behind general surgery, cardiac surgery, and orthopedics.^6^, ^8^, ^9^, ^11^, ^12^, ^13^ Yet unlike other subspecialties, no research has analyzed the current body of retracted publications in OHNS. This study sought to fill this gap in the literature and analyze the characteristics and trends of retracted publications in OHNS.

Our results showed a rise in retracted publications over time, with 79.2% of retracted publications being retracted during 2010 to 2022, compared to only 20.8% of retractions occurring during 1990 to 2009. Furthermore, we found that the average annual number of retracted publications and overall retraction rate for OHNS publications increased every decade since the 1990s. These trends have been found in previous studies on scientific literature.^8^, ^9^, ^10^, ^12^, ^13^ Wang et al found that 63% of retractions in Neurosurgery occurred within the last 5 years studied (2012‐2016) compared to 37% of retractions occurring from 1995 to 2011.^9^ Additionally, Rai and Sabharwal discovered there was a spike in the prevalence of retracted publications in Orthopedic Surgery in 2015 while analyzing retracted publications from 1984 to 2016.^8^

Several potential causes for the increase of retracted publications over time can be proposed. First, the overall rise in manuscripts being submitted can lead one to infer that the number of flawed manuscripts submitted could be rising over time as well. In addition, journals and reviewers are likely increasing review standards and meticulous review for plagiarism and misconduct, even after publication. These standards may have risen in part to the COPE guidelines being published in 2009, after which there was a significant increase in the annual retracted publication number in our study.^2^ These stringent standards have also been assisted by newly developed plagiarism‐detecting software such as Turnitin, which has been shown to be effective in deterring and identifying plagiarized works in academia.^9^, ^13^, ^14^ Furthermore, several scientists within the past decade have volunteered their time toward identifying fraudulent publications after being published that slipped through the journal's peer‐review process unscathed.^15^, ^16^ Additionally, as artificial intelligence (AI) continues to gain popularity to assist with a variety of tasks, one such recent use has been in detecting fraudulent or plagiarized publications. Most of the current AI software in this area has been focused on detecting duplication within figures and the publications themselves.^17^ While still in the early stages, several popular publishers of OHNS journals, including Wiley, Springer Nature, and Elsevier, have been experimenting with such software to screen submissions for duplication of images or text.^17^ As technology advances and AI continues to grow in use, it seems inevitable that fraudulent or flawed papers will increasingly be rejected, and the ones that make it to publication will be retracted soon after.

We found that the leading countries of origin for retracted publications were China, the United States, and Japan. Our results align with previous studies on retracted medical and scientific literature, with China, the United States, and Japan frequently among the top countries for retracted publication counts.^8^, ^9^, ^11^, ^18^ This can be in part due to the large number of submitted manuscripts from each of these countries, especially China, which has been the largest contributor in general science literature since 2016.^18^, ^19^ Furthermore, these countries have a long‐standing history of emphasis on research and pressure to publish in higher‐level schooling and academia in order to progress in one's career.^18^ While COPE guidelines have been in place, many journals are still implementing these guidelines, and research standards should be more stringent to preserve academic integrity and accuracy.

Plagiarism and duplicate publication were the 2 most frequent cited causes for retraction for the publications analyzed in our study. Our study aligns with previous work that has shown plagiarism to be the most common cause of retraction in other medical specialties.^7^, ^8^, ^9^, ^10^, ^11^ This finding indicates that academic dishonesty is implicated in a large number of retracted publications, an alarming and unfortunate finding in OHNS and other medical fields that may ultimately negatively impact patient care. For example, several retracted publications in this study by the same primary author looked at the most effective prophylactic antiemetic for patients who underwent different procedures and identified granisetron and ramosetron as most effective for thyroidectomy and pediatric tonsillectomy, respectively; these publications were retracted based on fraud, preventing what could have led to physicians citing these publications when giving these specific drugs as the first‐line prophylactic antiemetics.^20^, ^21^ With the rise of plagiarism‐detecting software, it is likely that many more papers will be caught and retracted in the short‐term due to plagiarism, but long‐term, there may be fewer retractions since most publications will be caught at the submission phase given increased access to such technology.

Finally, we found that the number of times a retracted publication was cited by other publications was statistically positively correlated with the journal's IF, which is an expected finding. The citation number was also positively correlated with the time a publication took to be retracted after initial publication. This seems to be a novel finding within the current body of literature. One would typically expect that a publication that has been cited many times would be under more immediate scrutiny by the academic community and ultimately retracted faster that less cited publications; however, the opposite was found in our study. The IF of a journal is unlikely to play a role in this since we found no significant correlation between IF and time to retraction. One theory for why this may occur is that when a publication has been cited multiple times, researchers may assume that the publication is strong and without major flaws. Another theory is the idea that a groundbreaking study with novel and statistically significant positive findings may be retracted after it is noticed and scrutinized by the scientific community, which can more frequently occur as a publication is increasingly cited. Regardless of cause, we should work to identify and retract flawed publications faster, since we found an overall mean publication to retraction time of nearly 3 years. Shortening this time period will help prevent these papers from being increasingly cited by other works.

Based on the results of our study, we can conclude that risk factors for a retracted publication in OHNS include China or the United States as the country of origin and clinical trials as the study type. While this is a novel finding in OHNS, similar risk factors can be seen for retractions in other surgical subspecialties. For Neurosurgery, over half of the retracted publications came from either China or the United States; however, the leading study types were basic science and reviews/meta‐analyses.^9^ Additionally, China and the United States consisted of the 2 most common countries of origin for retracted publications in Orthopedics.^8^ While not specifying clinical trials, 85% of retracted publications in Orthopedics were clinical studies.^8^ Retracted publications in Urology had similar risk factors; the United States and China had the most retracted publications, and randomized controlled clinical trials were the second most common study type after basic science studies.^10^ Given that these risk factors are shared across surgical subspecialties, journals, and editors may use this to increase the rigor of review for manuscripts with these risk factors. However, future studies may be done to determine whether these risk factors exist solely due to a large total volume of clinical trials and studies from China or the United States or if other associative factors may be at play, such as the social pressure to publish in these countries.

Our study is the first to analyze the characteristics and trends of retracted publications in OHNS. However, several limitations exist. First, this study looked at the top 60 journals in OHNS as determined by CiteScore. However, several ways to determine the top or most popular journals in the field exist, and a larger sample size could be obtained if more than 60 journals were used. In addition, since there is a mean of 3 years between publication and retraction, some of the most recent publications may not be retracted yet, and it, therefore, skews the results to underestimate the current number of recent retracted publications. Furthermore, some publications pertaining to OHNS may be published in journals outside of the 60 journals analyzed, underestimating the number of retracted OHNS publications. Additionally, studies were only included if they were listed in PubMed, were available in the English language, and had an abstract or full‐length publication available. This may limit us from utilizing other publications that did not fit these criteria. Finally, we classified publications into subtopics within OHNS and type of publication using the best judgment of multiple authors. Although multiple authors independently reviewing these will reduce bias, inherent human bias can still be present in these classifications.

## Conclusion

Our results demonstrate that retractions within OHNS journals have been increasing over time and have rapidly risen within the last decade. Furthermore, countries with long‐standing research‐centric traditions including China, the United States, and Japan were the most frequent origins for retracted publications. Plagiarism was the most common cause for retraction identified. Average time from publication to retraction was approximately 3 years, and this was seen to be positively correlated with the number of times the eventually retracted publication was cited by other papers. An increased understanding of common features of retracted publications in OHNS can assist journals, reviewers, and the rest of the academic community in preventing such publications from being published. If such publications are published, quicker retraction will benefit and maintain the integrity of the scientific community, and most importantly, benefit the patients. By remembering the primary goal of improving patient care over personal scientific and career advancement when conducting research, we would likely see a decline in fraudulent studies, ultimately leading to improved patient outcomes and increased trust in the OHNS community.

## Author Contributions

**Hannaan S. Choudhry**, design, data collection, data analysis and interpretation, writing, revision; **Sugosh M. Anur**, design, data analysis and interpretation, writing; **Hassan S. Choudhry**, design, data analysis and interpretation, writing; **Emily M. Kokush**, design, data analysis and interpretation, writing; **Aman M. Patel**, design, data analysis and interpretation, writing; **Christina H. Fang**, design, writing, review and editing, project supervision.

## Disclosures

### Competing interests

None.

### Funding source

None.

## Supporting information

Supporting information.

Supporting information.
