# Supplementary material for: Retracted Publications in Otolaryngology–Head and Neck Surgery: What Mistakes Are Being Made?
Source: OTO Open. 2024 Jun 13;8(2):e157. doi: 10.1002/oto2.157 (PMC11170335; doi:10.1002/oto2.157)
Supplement: Supplementary file 1 — Supporting information. [file OTO2-8-e157-s001.docx]

Supplemental Table 1. Top 60 Otorhinolaryngology journals as determined by 2021 Scopus CiteScore

| Rank | Journal |
| --- | --- |
| 1 | JAMA Otolaryngology – Head and Neck Surgery |
| 2 | Rhinology |
| 3 | International Forum of Allergy and Rhinology |
| 4 | Journal of Oral Pathology and Medicine |
| 5 | Ear and Hearing |
| 6 | Oral Diseases |
| 7 | Otolaryngology – Head and Neck Surgery |
| 8 | Trends in Hearing |
| 9 | Head and Neck |
| 10 | JARO – Journal of the Association of Research in Otolaryngology |
| 11 | Journal of Otolaryngology – Head and Neck Surgery |
| 12 | Clinical and Experimental Otorhinolaryngology |
| 13 | Archives of Oral Biology |
| 14 | Laryngoscope |
| 15 | International Journal of Oral and Maxillofacial Surgery |
| 16 | Dentomaxillofacial Radiology |
| 17 | Dysphagia |
| 18 | European Archives of Oto-Rhino-Laryngology |
| 19 | Journal of Cranio-Maxillo-Facial Surgery |
| 20 | Oral and Maxillofacial Surgery Clinics of North America |
| 21 | Sleep and Breathing |
| 22 | American Journal of Rhinology and Allergy |
| 23 | Clinical Otolaryngology |
| 24 | Medicina Oral, Patologia Oral y Cirugia Bucal |
| 25 | International Journal of Speech-Language Pathology |
| 26 | Journal of Oral Biology and Craniofacial Research |
| 27 | Head and Neck Pathology |
| 28 | Otolaryngologic Clinics of North America |
| 29 | American Journal of Speech-Language Pathology |
| 30 | Aphasiology |
| 31 | Audiology and Neurotology |
| 32 | Auris Nasus Larynx |
| 33 | Orthodontics and Craniofacial Research |
| 34 | Advances in Oto-Rhino-Laryngology |
| 35 | Acta Otorhinolaryngologica Italica |
| 36 | Journal of Vestibular Research: Equilibrium and Orientation |
| 37 | American Journal of Otolaryngology – Head and Neck Medicine and Surgery |
| 38 | Journal of Oral and Maxillofacial Surgery |
| 39 | Journal of Voice |
| 40 | Otology and Neurotology |
| 41 | Current Opinion in Otolaryngology and Head and Neck Surgery |
| 42 | European Annals of Otorhinolaryngology, Head and Neck Diseases |
| 43 | Head and Face Medicine |
| 44 | Laryngoscope Investigative Otolaryngology |
| 45 | Brazilian Journal of Otorhinolaryngology |
| 46 | Cranio – Journal of Craniomandibular Practice |
| 47 | Annals of Otology, Rhinology and Laryngology |
| 48 | British Journal of Oral and Maxillofacial Surgery |
| 49 | Cochlear Implants International |
| 50 | International Journal of Pediatric Otorhinolaryngology |
| 51 | Journal of Laryngology and Otology |
| 52 | Oral and Maxillofacial Surgery |
| 53 | Noise and Health |
| 54 | Journal of Audiology and Otology |
| 55 | Minerva Dental and Oral Science |
| 56 | ORL |
| 57 | Journal of Oral and Maxillofacial Pathology |
| 58 | Acta Oto-Laryngologica |
| 59 | Cleft Palate-Craniofacial Journal |
| 60 | International Archives of Otorhinolaryngology |
