# Supplementary material for: Retracted Publications in Otolaryngology–Head and Neck Surgery: What Mistakes Are Being Made?
Source: OTO Open. 2024 Jun 13;8(2):e157. doi: 10.1002/oto2.157 (PMC11170335; doi:10.1002/oto2.157)
Supplement: Supplementary file 2 — Supporting information. [file OTO2-8-e157-s002.docx]

Supplemental Table 2. List of 53 Otolaryngology – Head and Neck Surgery publications that were retracted during 1990-2022 and included in this study.

| Publication Title | Author and Year of Publication | Journal | Year of Retraction |
| --- | --- | --- | --- |
| Correlation of laryngeal and pharyngeal carcinomas and 24-hour pH monitoring of the esophagus and pharynx | Chen et al., 1998 | Otolaryngology - Head and Neck Surgery | 1999 |
| Low-dosage adrenaline induces transient marked decrease of blood pressure during functional endoscopic sinus surgery | Feng et al., 2006 | American Journal of Rhinology | 2006 |
| The effect of dental overbite on eustachian tube dysfunction in Iranian children | Azadani et al., 2006 | International Journal of Pediatric Otolaryngology | 2007 |
| Phonological mismatch makes aided speech recognition in noise cognitively taxing | Rudner et al., 2007 | Ear and Hearing | 2008 |
| The effect of nasal surgery on snoring | Elsherif et al., 1998 | American Journal of Rhinology | 2008 |
| Management of Congenital Incudostapedial Anomalies in Stapes Surgery. | Kuhn et al., 2007 | Otology & Neurotology | 2009 |
| What is the Role of Long-Term Macrolide Therapy in the Treatment of Recalcitrant Chronic Rhinosinusitis? | Soler et al., 2009 | The Laryngoscope | 2009 |
| When is Surgery Indicated for Asymptomatic Primary Hyperparathyroidism? | Morris et al., 2009 | The Laryngoscope | 2009 |
| Are Postoperative Hearing Results Better with Titanium Ossicular Reconstruction Prostheses? | Zeitler et al., 2009 | The Laryngoscope | 2009 |
| Modeling flow in a compromised pediatric airway breathing air and heliox [retracted in: Johnson J. | Mihaescu et al., 2009 | The Laryngoscope | 2009 |
| Regeneration of aged vocal fold: First human case treated with fibroblast growth factor. | Hirano et al., 2009 | The Laryngoscope | 2009 |
| Peroral endoscopic removal: as a minimally invasive long-term surgical treatment of a regurgitated giant polisegmented fibrovascular polyp of the esophagus | Iván et al., 2008 | European Archives of Oto-Rhino-Laryngology | 2010 |
| Minimally invasive peroral endoscopic removal of a regurgitated giant polysegmented fibrovascular polyp of the esophagus | Iván et al., 2008 | Dysphagia | 2010 |
| Management of obstructive sleep apnea in an edentulous patient with a combination of mandibular advancement splint and tongue-retaining device: a clinical report | Kurtulmus et al., 2008 | Sleep and Breathing: International Journal of the Science and Practice of Sleep Medicine | 2011 |
| Presurgical nasoalveolar molding with computer-aided reverse-engineering and rapid prototyping technique in infants with unilateral cleft lip and palate | Yu et al., 2011 | Journal of Oral and Maxillofacial Surgery | 2011 |
| Sinonasal Sarcoidosis | Lee et al., 2011 | Otolaryngology - Head and Neck Surgery | 2012 |
| Computed Tomography in 15 Congenital Aural Atresia Patients and Floating Mass Transducer on the Round Window | Carner et al., 2011 | Otolaryngology - Head and Neck Surgery | 2012 |
| Antiemetic Efficacy of Low-Dose Midazolam in Patients Undergoing Thyroidectomy. | Fujii et al., 2011 | Otolaryngology - Head and Neck Surgery | 2012 |
| Prevention of postoperative nausea and vomiting with antiemetics in patients undergoing middle ear surgery: comparison of a small dose of propofol with droperidol or metoclopramide | Fujii et al., 2001 | Archives of Otolaryngology-Head & Neck Surgery | 2012 |
| Anatomical variability of the maxillary artery: findings from 100 Asian cadaveric dissections | Kim et al., 2010 | Archives of Otolaryngology-Head & Neck Surgery | 2012 |
| Florid osseous dysplasia | Bansal et al., 2011 | Journal of Oral and Maxillofacial Pathology | 2012 |
| α4β1 integrin-dependent cell sorting dictates T-cell recruitment in oral submucous fibrosis | Rajendran et al., 2011 | Journal of Oral and Maxillofacial Pathology | 2012 |
| Selective use of hand and forearm muscles during bone screw insertion: a natural torque meter | Barros et al., 2012 | Journal of Oral and Maxillofacial Surgery | 2013 |
| erbB expression changes in ethanol and 7,12- dimethylbenz (a)anthracene-induced oral carcinogenesis | Jacinto-Aleman et al., 2013 | Medicina Oral Patologia Oral Y Cirugia Bucal | 2014 |
| Language abilities of patients with primary progressive multiple sclerosis: A preliminary group and case investigation | Barwood et al., 2013 | International Journal of Speech-Language Pathology | 2014 |
| A Prospective Study of the Surgical Outcome of Simple Uvulopalatopharyngoplasty (UPPP), UPPP Combined With Genioglossus Advancement or Tongue Base Advancement for Obstructive Sleep Apnea Hypopnea Syndrome Patients With Multilevel | Chen et al., 2015 | Clinical and Experimental Otorhinolaryngology | 2016 |
| Laryngeal Function After Radiation Therapy | Gamez et al., 2015 | Otolaryngologyic Clinics of North America | 2017 |
| Prevention of vomiting after tonsillectomy in children: granisetron versus ramosetron | Fujii et al., 2001 | The Laryngoscope | 2017 |
| Granisetron, droperidol, and metoclopramide for preventing postoperative nausea and vomiting after thyroidectomy | Fujii et al., 1999 | The Laryngoscope | 2017 |
| Prevention of nausea and vomiting after middle ear surgery: granisetron versus ramosetron | Fujii et al., 1999 | The Laryngoscope | 2017 |
| False aneurysm of the facial artery as complication of sagittal split osteotomy | Pappa et al., 2008 | Journal of Craniomaxillofacial Surgery | 2017 |
| Interleukin-33 promotes helper T cell type-2/17 inflammation in children with allergic rhinitis | Wang et al., 2014 | European Archives of Oto-Rhino-Laryngology | 2017 |
| Microarchitecture and Biomechanical Evaluation of BoneCeramic Grafted Alveolar Defects during Tooth Movement in Rat | Ru et al., 2016 | The Cleft Palate-Craniofacial Journal | 2017 |
| Dexamethasone for the prevention of recurrent laryngeal nerve palsy and other complications after thyroid surgery: a randomized double-blind placebo-controlled trial | Schietroma et al., 2013 | JAMA Otolaryngology Head Neck Surgery | 2018 |
| Generic Quality of Life in Persons With Hearing Loss: A Review of the Recent Literature | Brodie et al., 2018 | Otology & Neurotology | 2019 |
| Dexamethasone injection into the pterygomandibular space in lower third molar surgery | Boonsiriseth et al., 2017 | International Journal of Oral and Maxillofacial Surgery | 2019 |
| A safe and accurate method to perform esthetic mandibular contouring surgery for Far Eastern Asians | Hsieh et al., 2016 | International Journal of Oral and Maxillofacial Surgery | 2019 |
| Propofol alone and combined with dexamethasone for the prevention of postoperative nausea and vomiting in adult Japanese patients having third molars extracted | Fujii et al., 2007 | The British Journal of Oral & Maxillofacial Surgery | 2019 |
| Estimation of salivary and serum alkaline phosphatase level as a diagnostic marker in type-2 diabetes mellitus with periodontal health and disease: A clinico-biochemical study | De et al., 2018 | Journal of Oral and Maxillofacial Pathology | 2019 |
| Voice Quality in Adults Treated for Unilateral Cleft Lip and Palate: Long-term Follow-up After 1- or 2-Stage Palate Repair | Morén et al., 2019 | The Cleft Palate-Craniofacial Journal | 2019 |
| Conservative management of subglottic stenosis with home based tracheostomy care: A retrospective review of 28 patients | Pelser et al., 2018 | International Journal of Pediatric Otolaryngology | 2020 |
| Management of seborrhoeic keratosis and actinic keratosis with an erbium:YAG laser-experience with 547 patients | Sayan et al., 2018 | International Journal of Oral and Maxillofacial Surgery | 2020 |
| Coronavirus disease-2019: A brief compilation of facts | Saxena, 2020 | Journal of Oral and Maxillofacial Pathology | 2020 |
| Neoadjuvant chemotherapy as a comprehensive treatment in patients with laryngeal and hypopharyngeal carcinoma | Wei et al., 2020 | Acta Oto-Laryngologica | 2020 |
| Outcome Measurement in the Treatment of Spasmodic Dysphonia: A Systematic Review of the Literature | Rumbach et al., 2019 | Journal of Voice | 2021 |
| Does Low-Level Laser Photobiomodulation Improve Neurosensory Recovery After Orthognathic Surgery? A Clinical Trial With Blink Reflex | Haghighat et al., 2021 | Journal of Oral and Maxillofacial Surgery | 2021 |
| Performance comparison of vibration devices on orthodontic tooth movement - A systematic review and meta-analysis | Keerthana et al., 2020 | Journal of Oral Biology and Craniofacial Research | 2021 |
| Primary orbital reconstruction with selective laser melted core patient-specific implants: overview of 100 patients | Rana et al., 2019 | The British Journal of Oral & Maxillofacial Surgery | 2021 |
| Minimal Scar Dissection for Partial Parotidectomy via a Modified Cosmetic Incision and an Advanced Wound Closure Method | Zhang et al., 2019 | Journal of Oral and Maxillofacial Surgery | 2022 |
| Active Versus Passive Transcutaneous Bone Conduction Hearing Devices: A Systematic Review and Meta-Analysis | Forner et al., 2022 | Ear and Hearing | 2022 |
| Neuromuscular Specializations of the Human Hypopharyngeal Muscles | Mohammed et al, 2020 | Dysphagia | 2022 |
| Variations in the labyrinthine segment of facial nerve canal revealed by high-resolution computed tomography | Jin et al., 2017 | Auris, Nasus, Larynx | 2022 |
| Baicalin exerts anti-tumor effects in oral squamous cell carcinoma by inhibiting the microRNA-106b-5p-Wnt/β-catenin pathway via upregulating disabled homolog 2 | Wang et al., 2021 | Archives of Oral Biology | 2022 |
